# Readiness for Delivering Digital Health at Scale: Lessons From a Longitudinal Qualitative Evaluation of a National Digital Health Innovation Program in the United Kingdom

**DOI:** 10.2196/jmir.6900

**Published:** 2017-02-16

**Authors:** Marilyn R Lennon, Matt-Mouley Bouamrane, Alison M Devlin, Siobhan O'Connor, Catherine O'Donnell, Ula Chetty, Ruth Agbakoba, Annemieke Bikker, Eleanor Grieve, Tracy Finch, Nicholas Watson, Sally Wyke, Frances S Mair

**Affiliations:** ^1^ Digital Health and Wellness Group Computer and Information Sciences University of Strathclyde Glasgow United Kingdom; ^2^ General Practice and Primary Care Institute of Health and Wellbeing University of Glasgow Glasgow United Kingdom; ^3^ School of Health and Social Care Edinburgh Napier University Edinburgh United Kingdom; ^4^ Health Economics and Health Technology Assessment Institute of Health and Wellbeing University of Glasgow Glasgow G12 8RZ, UK United Kingdom; ^5^ Institute of Health and Society Newcastle University Newcastle Upon Tyne NE2 4AX United Kingdom; ^6^ School of Social and Political Sciences Institute of Health and Wellbeing University of Glasgow Glasgow United Kingdom

**Keywords:** telemedicine, health plan implementation, community health services, health services research, electronic health records, instrumentation, qualitative research, diffusion of innovation, medical informatics

## Abstract

**Background:**

Digital health has the potential to support care delivery for chronic illness. Despite positive evidence from localized implementations, new technologies have proven slow to become accepted, integrated, and routinized at scale.

**Objective:**

The aim of our study was to examine barriers and facilitators to implementation of digital health at scale through the evaluation of a £37m national digital health program: ‟Delivering Assisted Living Lifestyles at Scale” (dallas) from 2012-2015.

**Methods:**

The study was a longitudinal qualitative, multi-stakeholder, implementation study. The methods included interviews (n=125) with key implementers, focus groups with consumers and patients (n=7), project meetings (n=12), field work or observation in the communities (n=16), health professional survey responses (n=48), and cross program documentary evidence on implementation (n=215). We used a sociological theory called normalization process theory (NPT) and a longitudinal (3 years) qualitative framework analysis approach. This work did not study a single intervention or population. Instead, we evaluated the processes (of designing and delivering digital health), and our outcomes were the identified barriers and facilitators to delivering and mainstreaming services and products within the mixed sector digital health ecosystem.

**Results:**

We identified three main levels of issues influencing readiness for digital health: macro (market, infrastructure, policy), meso (organizational), and micro (professional or public). Factors hindering implementation included: lack of information technology (IT) infrastructure, uncertainty around information governance, lack of incentives to prioritize interoperability, lack of precedence on accountability within the commercial sector, and a market perceived as difficult to navigate. Factors enabling implementation were: clinical endorsement, champions who promoted digital health, and public and professional willingness.

**Conclusions:**

Although there is receptiveness to digital health, barriers to mainstreaming remain. Our findings suggest greater investment in national and local infrastructure, implementation of guidelines for the safe and transparent use and assessment of digital health, incentivization of interoperability, and investment in upskilling of professionals and the public would help support the normalization of digital health. These findings will enable researchers, health care practitioners, and policy makers to understand the current landscape and the actions required in order to prepare the market and accelerate uptake, and use of digital health and wellness services in context and at scale.

## Introduction

It is often the case with eHealth and digital health studies that 1 single service or product is studied in a controlled setting (often a randomized control trial) to determine its effectiveness in order to proceed to roll it out at scale and make it part of routine care delivery pathways. Previous research, however, has shown that uptake and adoption are slow for digital health overall and that there may be many sociotechnical, organizational, or cultural barriers that are slowing the mainstreaming of digital health [1,2]. Over the last two decades, there has been an exponential growth in the literature describing barriers and facilitators to innovation [3] and digital health implementation [4]. This literature initially focused on examining implementation issues on a relatively small scale [5,6] but this was followed by more extensive studies looking at large scale deployments of single digital health services in particular contexts [7-11]. Issues that have been identified as barriers to implementation range from liability concerns, interoperability issues, costs, usability, misaligned incentives, and policy problems through to acceptability to patients and professionals [4,8].

The concept of ‟eHealth readiness” has previously been highlighted [12,13] and there is increasing interest in using tools to better measure specific aspects of digital health readiness [14-16]. However, the existing evidence mainly relates to organizational readiness within the health service [17,18] or the readiness of health professionals [19-21], patients or carers for a specific single digital health service [22]. The technical, political, and market preparedness and readiness for widespread delivery of consumer-oriented digital health services which encompass and cross health-social-technological boundaries has not yet been fully explored. Consequently, a ‟whole systems” analysis of readiness for digital health is warranted.

Delivering Assisted Living Lifestyles at Scale (dallas) was an ambitious national program conducted from May 2012 to May 2015 in the United Kingdom. The program received £37m ($55m, €50m) in funding from Innovate UK, the National Institute for Health Research (NIHR), The Scottish Government, Scottish Enterprise, and Highlands and Islands Enterprise. The dallas program aimed to develop and implement a wide range of digital health and wellness products and services to enable preventive care, self-care, and independent living at scale. One of the program’s primary goals was to stimulate the consumer market for person-centered digital technologies. It was explicitly set up as a large scale research and development program rather than a randomized clinical trial or a series of individual pilots. This was considered crucial by the program funders to begin to understand what the existing barriers to uptake and adoption of digital health at scale are and to unlock new markets and pathways to make digital health at scale a reality.

Four large multi-agency consortia (referred to as communities) called ‟i-Focus,” ‟Living It Up,” ‟More Independent,” and ‟Year Zero” were funded (see Multimedia Appendix 1 for details of each consortium). The communities were funded specifically to design, deploy, and promote awareness and uptake of a range of innovative digital health and wellness services across the United Kingdom. Innovation and ability to scale up and sustain digital health and open new routes to market were considered key to the program’s success. Unlike many other previous digital health trials or studies, the services developed were aimed at a broad sociodemographic, including children, parents, older adults, as well as the broader consumer health and fitness population living in urban or remote and rural regions. Some of the services were digital and aimed at increasing awareness, redesigning services or care pathways, as well as increasing the uptake and routinization of digital health as a whole. Details of these consortia have been reported previously [23], but Multimedia Appendix 1 briefly summarizes the wide range of stakeholders involved in each consortium and the range of digital health products, services, and activities developed and delivered via the dallas program.

Due to the variety of products and services (apps, personal health records, telecare, telehealth, wearable activity trackers, and so on) and the variety of populations and contexts, communities were measuring a range of traditional primary outcomes such as levels of engagement, perceived usability and acceptability of the products, and reduction in resource usage (such as hospitalizations). In addition to this, however, the communities were exploring ways to capture changes (positive increases) in the amount and quality of choice, contribution, community, collaboration, and connectedness that the new services created.

In this paper, we present synthesized qualitative findings from a longitudinal study of digital health design, delivery, and roll out. We examine implementation issues from different angles and with different stakeholders with a focus on what this data tells us about the readiness of different elements of the ecosystem in the United Kingdom to deliver digital health at scale. Given the current self-care agenda, the drive toward more personalized medicine [24,25], and person-centered digital health solutions [26,27], such work is timely and has the potential to make an important contribution to understanding the implementation of digital health innovations internationally. The aim of this study was to capture barriers and facilitators to implementation of digital health across a wide range of stakeholders and across time, thus allowing us to answer the question of how ‟ready” different people, processes, and systems are for mainstreaming digital health and to identify what measures might be taken to reduce some of the existing and persistent barriers in this area. Here we present our findings and conclude with a set of 10 recommendations to address some of the key readiness barriers identified.

## Methods

### Aim and Design

Longitudinal qualitative and survey data were collected over 39 months (June 2012-October 2015) to help us identify and understand key barriers and facilitators experienced during the implementation journey. Tables 1 and 2 illustrate the full breadth and volume of data collected.

**Table 1:** Qualitative implementation dataset (interviews).

Interviews		Number of items	Number of participants	Number of pages
**eHealth Implementation Toolkit (e-HIT) interviews**		47	53	1134
	Interviews: Baseline e-HIT Dates: October 2012-January 2013 Participants: 7 health, 6 industry, 3 voluntary, 2 academia Dallas community: iF, LiU, Mi, YZ	17	18	247
	Interviews: Midpoint e-HIT Dates: October 2013-December 2014 Participants: 13 health, 7 industry, 3 voluntary, 1 academia Dallas community: iF, LiU, Mi, YZ	20	24	630
	Interviews: Endpoint e-HIT Dates: May-October 2015 Participants: 5 health, 5 industry, 1 voluntary sector Dallas community: iF, LiU, Mi, YZ	10	11	257
**Champions**		22	23	233
	Interviews: Lay champions Dates: December 2013-December 2014 Participants: 11 volunteer champions, 2 voluntary sector, 1 health service, 2 administrator, 1 other Dallas community: Mi, LiU	17	17	186
	Interviews: Digital champions Dates: March 2015 Participants: 5 voluntary champions, 1 government Dallas community: Mi	5	6	47
**LiU stakeholders**		32	20	545
	Interviews: LiU longitudinal interviews Dates: January 2014-January 2015 Participants: 5 health service managers; 1 health service lead Dallas community: LiU	18	6	315
	Interviews: LiU cross-sectional interviews Dates: July 2014-April 2015 Participants: 2 health prof, 6 industry, 2 voluntary sector, 2 government, 1 academia, 1 consumer user Dallas community: LiU	14	14	230
**Project management or cross project**		24	26	248
	Interviews: Evaluation alignment Dates: May-November 2014 Participants: 1 health service, 4 industry Dallas community: iF, Mi, YZ	5	5	11
	Interviews: dallas leads Dates: June 2015 Participants: 3 health service, 2 industry Dallas community: iF, LiU, Mi, YZ	5	5	46
	Interviews: Digital Health And Care Alliance Dates: March-April 2015 Participants: 1 health, 4 industry, 1 academia, 1 voluntary, 3 government Dallas community: iF	10	10	168
	Interviews: House of Memories Dates: July-October 2015 Participants: 2 patients, 2 carers, 1 industry, 1 government Dallas community: Mi	4	6	23
Subtotal		125	122	2,160

**Table 2:** Qualitative implementation dataset (focus groups).

Focus groups	Number of items	Number of Participants	Number of pages
Focus group *:* Lay champions Dates: December 2013 Participants: 8 volunteer champions Dallas community: Mi	1	8	23
Focus group: House of Memories Dates: March 2015 Participants: 4 patients, 4 carers, 1 health service and 1 government agency staff Dallas community: Mi	1	10	40
Focus group: eRedBook Dates: April 2015 Participants: 12 health service users, 9 health professional users, 2 health service; 2 industry Dallas community: YZ	2	25	71
Focus group: No delays Dates: April 2015 Participants: 4 health service users, 4 health service staff & 1 administrator Dallas community: YZ	2	9	49
Focus group: Get active Dates: May 2015 Participants: 5 users & 2 voluntary sector staff Dallas community: LiU	1	7	19
Subtotal	7	59	202

Detailed evidence was gathered from numerous stakeholders rolling out different services to enable a rich understanding of digital health readiness (see Tables 1 and 2). From this, detailed reports describing the diverse experiences of each group within their context, the process of rolling out products or services, and factors that shaped each consortium’s implementation journey were written. Cross-case analysis of communities was conducted, drawing out not only commonalities related to ‟readiness” but also differences or alternative explanations of factors affecting readiness for digital health at the individual, organizational, or wider environmental and political level.

### Sampling and Setting

Specific roles and partner organizations were identified by the research team as critical to capturing perspectives of each stakeholder group within the consortia. This was wide ranging and included health care professionals; health and social service managers; staff and volunteers from third sector organizations; private companies designing, developing, and promoting hardware and software platforms; and academics and employees from government agencies working on guidelines and policies. Geographic locations spanned England and remote rural and urban regions of Scotland. Care was taken to include representatives from all types of organizations involved (private, public, voluntary) and to include stakeholders involved at the strategic level, the project management level, and the service design and delivery level. Progress of the program was followed longitudinally over 3 years. Interviews were undertaken with different stakeholders over this period. A subset of key individuals was subjected to repeat interviews, for example, as part of our eHealth Implementation Toolkit (e-HIT) interviews and our case study work, allowing us to access the perspectives of key implementers at different points during the program.

### Data Collection and Management

Perspectives of key implementers from each community were sought via semistructured interviews. The structure of the interview was based on expanding on issues raised when using the e-HIT tool at baseline, midpoint, and end of the program [28]. This tool was developed in previous research by members of the team [28] and is designed to help promote understanding of digital health implementation issues. Focus groups were held with end users including patients, carers, consumers, health professionals, and local champions (see Table 2). Interviews and focus groups were audio-recorded with participant consent, transcribed verbatim, and anonymized. This dataset was supplemented with reflective notes and ethnographic data noted during primary data collection. Electronic and paper-based survey data collection was also undertaken to gather opinions from health professionals.

### Theoretical Approach

Increasingly, in implementation research, the use of theory has been advocated in order to allow us to develop an improved understanding and explanation of why service innovations or digital health technologies become an integrated part of routine service delivery or not [29]. A range of theories have been utilized such as actor network theory [30] through to theories of organizational readiness [31].

Our evaluation was underpinned by a sociological theory, normalization process theory (NPT) [32,33], which has been used extensively to enhance understanding of how individuals and groups of people understand, integrate, and sustain new technologies, service innovations, or ways of working into everyday practice [32]. NPT has 4 core constructs (Multimedia Appendix 2) and is the underpinning for the 2 instruments used as part of the evaluation toolkit. The e-HIT [28] was used for 47 qualitative stakeholder interviews undertaken over the 3 years of the project (baseline, midpoint, and end point, see Table 1) and the NoMAD [34] questionnaire, derived from NPT constructs, was used with 48 health professional respondents involved with the No Delays Service. One community (Living it Up) was also studied more widely across stakeholders and across time as a case study.

NPT provided a consistent and coherent theoretical lens to analyze and interpret data across the program which enabled us to systematically identify themes and to provide structure to any explanation we could identify in the data for the emerging key themes. Our use of NPT as the theoretical underpinning of our analysis across the dallas program has allowed us to use NPT as a lens through which to conceptualize data at different levels, thereby taking into account wider contextual and environmental factors as well as workability issues at the individual level. It has therefore helped us to make propositions or recommendations for future large scale digital health implementation programs.

### Data Analysis

Qualitative data analysis was informed by a framework approach [35], using a coding frame informed by NPT [23]. The data (interviews, project documentation, field notes) were presented as transcripts and notes and the text coded (tagged within qualitative data software called NVivo (QSR International) to generate an annotated coding book. Coding clinics were undertaken to ensure consistency of coding and shared understanding of coding constructs. A total of 8 researchers with multidisciplinary experience in health services research, clinical research, informatics, and social science were involved at coding clinics with 1 or 2 senior academics (FSM, MML) involved in all coding sessions. Care was taken to expand, collapse, and rename codes so that all were confident that the coding book was a fair and accurate reflection of the data. Integrative analyses and key mapping of emerging themes undertaken during the final phase of the project identified ‟readiness” concepts as a key theme visible across the NPT framework. A matrix of overarching readiness themes was then coded in parallel with our NPT framework (see Table 3). Issues falling outside the NPT framework, like ‟organizational culture,” were noted.

### Ethics and Governance

This evaluation was commissioned by Innovate UK. University ethical approvals were granted for the collection of all qualitative data reported (College of Medical, Veterinary and Life Sciences, Approval: 200130141; and College of Science and Engineering, Approval: CSE01210 and CSE01096 at the University of Glasgow as well as University of Newcastle ethics approval Reference number 00555/2012). Our work was overseen by an external advisory group. Informed consent to participation was gained from all participants. Anonymity was protected by use of generic descriptors throughout.

## Results

### Analysis and Coding Scheme

The first significant result to report is the breadth and volume of data collected across the stakeholders via the different mixed methods. Table 1 in the methods section provides an overview that helps to contextualize where our results, discussed in the following section, emerge from. We next present an overview of the final coding structure with discussions and exemplar quotes from each emerging theme. A more detailed set of representative quotes per theme can be found in Multimedia Appendix 3.

**Figure 1:**
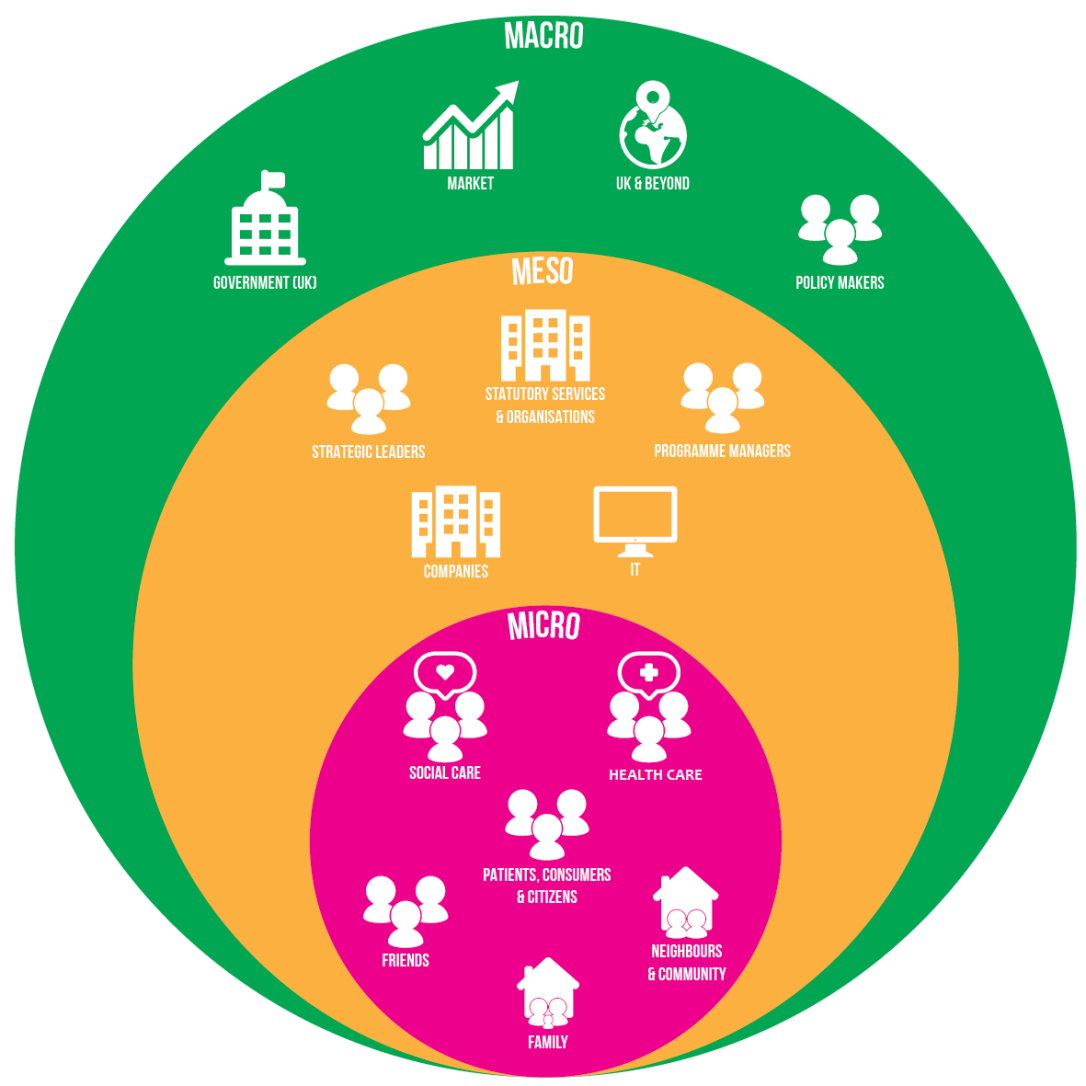
Key themes influencing readiness for digital health.

Our analysis revealed that readiness issues were present at 3 levels (Figure 1): Macro (market, policy or governmental, wider context), meso (commercial, organizational, and infrastructure), and micro (professional, public, carers).

The full coding scheme is illustrated in Table 3 together with how it maps onto our NPT theoretical framework.

**Table 3:** Overview of readiness coding scheme mapped to normalization process theory (NPT) constructs.

Level	Theme
**Macro**	
Market	Interoperability (collective action)
	Risk or liability (coherence and collective action)
	Clinical endorsement (collective action)

Policy or infrastructure	National policy (collective action)
	Infrastructure (collective action)
**Meso**	
Industry	Incoherent market (coherence, cognitive participation)

National Health System (eg, National Health Service)	Information technology infrastructure (collective action)
	Discontinuity and organizational culture (some collective action or cognitive participation but some outside normalization process theory)
	Resources (collective action)
**Micro**	
Health professional readiness	Workload and professional confidence (collective action)
	Training & alignment with professional roles or identity (collective action)
	Access to digital resources (collective action)
Public readiness of digital health services and systems	Digital literacy and access (cognitive participation or collective action)
	Agency and lifestyle (coherence)
	Security and trust (collective action)

The key issues (those that were most prevalent in the data) under each of these main ‟readiness” themes are now described in detail with illustrative quotations provided to support each key point (see Multimedia Appendix 3 for additional supporting quotes). The discussion section goes on to present recommendations based on these findings which we argue will accelerate the uptake, mainstreaming, and ultimate success of digital health at scale.

### Macro-Level Readiness

As described in our overarching approach, it was considered critical to examine not just single products or geographies in localized contexts but to also use a wider lens to capture barriers or facilitators to digital health at the full ecosystem level. This includes the systems and structures socially, technically, and politically that are needed, and how ready these systems are to support and even promote digital health. Two key overarching themes were identified at the macro level: ‟market readiness” and ‟national policy or infrastructure readiness.” They are presented here with each of their subthemes with representative quotes from the data.

#### Market Readiness

One of the program’s primary goals was to stimulate the consumer market for person-centered digital technologies. This was identified as an ongoing tension and required continual navigation and negotiation during the life of the program. Four key areas contributed to this challenge at the time of writing and are described in turn here.

#### Interoperability

Interoperability is a key issue for digital health products and services, particularly when they have to interface and exchange data with National Health Service (NHS) and other clinical and social care information systems. To address this, 1 consortium, i-Focus, invested significant time and resource developing a reference architecture which could serve as a common technical framework to work across sectors. This consortium also established the Digital Health and Care Alliance (DHACA), a not-for-profit, member-driven organization, building understanding and sharing knowledge and expertise between small- and medium-sized enterprises and the statutory sector on how to develop and implement interoperable digital health and wellness services.

Our data revealed that interoperability was perceived as more than a technical challenge. Commercial companies often perceive open standards and interoperability as a threat to their business model, since it was not a priority in comparison with efforts to increase their own individual market share as the market develops and matures. Lack of interoperability was a clear barrier to progress.

...there is an element of maybe we could work more together, but where do I spend my time? Do I spend my time in a meeting discussing data integration with another company when I've got only tens of thousands users worldwide, or do I try and get into users worldwide then worry about it?

DHACA interviewee no. 2, 2015

#### Risk and Liability

Providing digital health services for people with major frailty or multimorbidity was still perceived as risky in terms of product liability issues by some commercial companies. Concerns were raised by industry partners—especially those that traditionally operate outside health and social care—about the responsibilities entailed by operating within the digital health statutory sphere.

It’s a young person’s market, or it’s a ‟worried well” market. So the people who buy fitbit and pedometers. And that's great, (...) because they are responsible for the outcome of that monitoring. If you’re monitoring a heart condition, you want someone at the end of that. You don’t just want a fitbit, you want a Triage Nurse. So...I’m not entirely convinced that there is a consumer market in these technologies, actually...I think there's a hybrid market, maybe,...And I think we might be a way off, you know, finding it...it is really complex.

DHACA interviewee No. 9, 2015

Providing self-monitoring devices as part of health promotion was deemed acceptable but providing clinical data back to those with known health problems was perceived as a different proposition, involving significant risk. Companies often wanted to sell a ‟technology kit,” whereas the statutory sector wanted a different type of ‟contract,” linking the use of technology to data services and outcomes. This was a risk which companies were reluctant to take on in the current market until it was clear what the ethical guidelines and responsibilities were around collection and use of lifestyle data and was another barrier to implementation.

#### Clinical Endorsement

Accreditation and clinical endorsement were seen as crucial issues affecting deployment plans for digital health. Clinical endorsement could involve a single health professional endorsing it to people in their practice for example, or more likely a body of clinicians publically backing up or signing up to say that they think that the product is useful and clinically beneficial. This can still have a huge effect on digital health product success—even if that product is meant to appeal to the consumer staying well, as opposed to a patient being treated for a condition.

A purely consumer version of the eRedBook, a digital child health record created as part of the Year Zero consortium, did not prove viable initially. This was because endorsement by the relevant medical association was seen as a prerequisite to ensure uptake by lay users and health professionals; obtaining such accreditation was labor and time intensive and posed a barrier for implementation but once accreditation or endorsement was achieved, this was perceived as a potential facilitator of uptake.

What our experience brought us to realize is that people will only use a personal health journal around serious or long term conditions if it’s something they can engage with their clinicians on. You’re not going to persuade people to go out and buy it as a consumer product if it’s not something their clinicians will engage with them on and look at and share the information that they've been collecting.

C3 Implementer interview, June 2015

Accreditation and official endorsement of digital health products and services were seen as key ongoing issues likely to influence deployments and future development of digital health, and further research and policy work is required to clarify what apps and services require accreditation (and which do not) and also what such accreditation should look like.

#### Complexity of the Market

The digital health market proved difficult to access and navigate especially for international companies or start-ups unfamiliar with the landscape. The organization and delivery of health care is currently devolved to the 4 countries of the United Kingdom. Each individual health service is composed of a large number of heterogeneous and autonomous organizations functioning in substantially distinct ways. The interface between health and social care varies and many products and services are now more lifestyle or wellness based and not clearly and solely within the remit of either health or social care uniquely. All of this makes the UK digital health market challenging to navigate, with a lack of clear access or entry points for the retail sectors. The eclectic nature of the dallas consortia helped provide opportunities for people to connect and learn how to navigate such a complex landscape and to experiment with different models and pathways to implement and mainstream digital health that might not have been considered traditionally.

...the amount of red tape from the National Health Service and the...finance committees, procurement committee, it would have been very difficult to know who would be the right person to speak to so it’s kind of opened opportunities for people to get round the table and have real discussions about how they can make a difference and that’s been a really positive part of the program.

Final e-HIT interview C2, interview 1, social care manager

#### Political Readiness and National Policy

Information governance policies and legislation issues within the health and social care sectors were a recurring theme. Regulations around information governance generally are strongly embedded and well established in the UK health service due to its culture of high security, with patient confidentiality viewed as a priority. Although policy and legislation relating to data sharing has been reviewed and clear recommendations made [36], this has not yet translated to local contexts and was reported as a key barrier to deployment of the dallas program across its lifetime.

There is a real problem...health data is in a vault that’s owned by the National Health Service. You can’t, at the moment, view it and when you can view it, it will be a view which is not in a form that can be used by technology outside of the NHS in any real useful way. I think the biggest issue is information governance and letting people take ownership of their own data and their own risk appetite, and until that happens all we are doing is allowing the market to develop outside of the true record.

DHACA interview 3, 2015

The notion of sharing sensitive health data across multiple public and private organizations that do not hold the same information governance rules is fraught with difficulty. This led to a common view that information governance regulations were not currently ‟fit for purpose.” If we are trying to change existing care paradigms, responsibilities, and data ownership for digital health, it is clear that further work is needed around specific information governance for health and wellness products that are not covered by existing clinical or statutory policy or governance. For example, accessing ‟Twitter” or similar sites using hospital computer systems was often not permissible, which meant that integrating social networking platforms within the health care arena was problematic.

Some recent national policies were seen as positive drivers for change among certain consumer groups. For example, the experience from digital enablement activities suggested that recent social benefits reforms compelled people ‟to sign on the Web.” This encouraged many individuals to improve their computer literacy by joining ‟Lay” and ‟Digital champion” programs and to engage with digital hubs so they could access their social welfare benefits. As a result, disadvantaged groups potentially at risk of digital exclusion were provided with digital skills and educated about health technologies. Policy and funding streams need to advocate and support digital inclusion and awareness-raising if digitally supported self-management is to become a reality for people on the ground accessing the services.

...the benefits reform has been a great carrot or stick to push people...you’ll hear stories from Digital Champions, people coming in on a Friday afternoon because they are going to get sanctioned if they don't do this online form...

Digital champion interview, March 2015

#### Infrastructure

Our data showed significant variation in national infrastructure across the United Kingdom. Those in remote and rural areas voiced concerns about inadequate Internet connectivity as a limiting factor for accessing digital health services. Health centers in urban areas also reported to lack the connectivity necessary to enable access to new digital health services being rolled out. Organizations at the local and national level clearly need to invest in information technology (IT) infrastructures if digital services are to be rolled out and supported robustly across the United Kingdom.

Area (X) did phenomenally well given the poor connectivity in the region you know poor WiFi and even when we 3G-enabled their tablets poor 3G signals you know, it was a hard slog of going around x centers and signing people up.

C3 final e-HIT interview 1

### Meso-Level Readiness

At the intermediate or ‟meso” level there were 2 main themes identified: ‟Industry” and ‟local health service organizational” readiness. These are related to the specific markets and organizations required to access and roll out digital health.

#### Industry Readiness

Digital health is constantly promoted as a potentially lucrative market. However, enticing commercial entities—who normally sell products directly to consumers—to invest in opportunities in emerging digital health, wellbeing, or social care sectors did not prove as straightforward as originally anticipated in the dallas program. This may be due in part to lack of a coherent market at the time of the program.

#### Lack of Market Coherence

Market stability and maturity were key themes for industry readiness. Private industries that normally operate outside of health and social care were reticent to engage with the relatively immature, digital health sector, proving less ‟ready” to invest than anticipated. One community tried hard to engage with well-respected retailers but found it impossible to translate initial interest into actual delivery of digital health offerings. In addition, some private industries did not fully grasp what consumers wanted or required of them in terms of digital health products.

…I think user experience is key so start with the language of the consumer and language of the value proposition, people who are selling to consumers who are parting with their money rather than looking at the language of local authorities and health sector which is all about cost avoidance…

C4 implementer interview, June 2015

#### Collaboration, Competition, and Codesign

Codesign methodologies and intensive consumer engagement were successfully utilized in the program at scale to address knowledge gaps in consumer preferences. This work reinforced the view that a one size fits all approach would not work. However, the time and effort required for this created real challenges, as it introduced delays and consequently reduced the time available to develop and deploy new solutions within the defined timescale of the program.

‟Collaboration versus competition” were also key themes. Some private industries were understandably very protective of their intellectual property, which made them unwilling to share expertise and technology solutions with third parties in a large multi-stakeholder environment.

...It’s a sort of codesign, and what happens is people take a long time to make up their mind which compresses time for technical partners (...) for some people working in a collaborative nature with technical partners is a new environment so they are cautious and wary of telling all their secrets in case people run away with them so I think there is a protective defensive mode.

C1 (Midpoint), e-HIT interview

#### Health Service Readiness or Information Technology (IT) Infrastructure

The variation in workflow processes and in-house IT and data management systems—which stem from the historical foundations of hospitals operating as separate entities within a confederated health system—continues to impede the advancement and integration of digital health initiatives.

‟Technical readiness” was often an acute issue at local organizational level as legacy systems, firewalls, and strict information security procedures within health boards, hospital trusts, health centers, or general practitioner (GP) practices varied from site to site and left health professionals and implementers ill-equipped to readily deploy solutions implemented elsewhere. This became apparent as digital platforms designed and developed in a specific context could not be rolled out elsewhere at scale due to lack of process and technical standardization across the UK health services.

Well things like legacy systems, fire walls, when we are adopting new technologies, eHealth capacity, eHealth priorities within the internal infrastructure is stretched.

C1, Final e-Hit interview 1

Systems interoperability and lack of integration was a recurrent issue as computer systems across the health and social care sector could not easily exchange data with applications created as part of dallas. Practically this meant that each new implementation site had to go through its own deployment ‟pain-barrier,” with ad-hoc local solutions providing little insight on what to expect elsewhere. This limited the effectiveness of many of the new digital platforms and required workarounds by health care staff and end users to ensure the benefits could still be exploited locally.

#### Discontinuity and Organizational Culture

The restructuring of the health service in England meant it was particularly difficult to get health service partners to maintain focus and be ready to deliver aspects of the program they had originally signed up to as there were so many changes taking placing concurrently with the roll out of the dallas program. Constant change made ownership and responsibility for digital health services unclear and a lack of senior management buy-in was also cited as a barrier to organizational readiness for digital health, which could negatively affect implementation efforts. This recurring flux and uncertainty had a knock-on effect in other areas.

Some health organizations had not yet fully developed their own digital strategies and/or had not yet bought in fully to the self-care agenda at the time of the program.

It’s people who have to implement that need to move in a different direction to achieve what we need to achieve...It’s such a complex system that you can’t simply commission eHealth technology, it can’t be done! There are too many stakeholders who could block, misunderstand or not want to get involved...

C2 (Midpoint) e-HIT interview 5

It became clear that in order to be successful, digital health innovation must be closely aligned with health service organizational vision and road maps for change. Problems at the executive level within larger health trusts were contrasted with readiness of smaller, more flexible organizations such as general practices, which seemed more receptive to adopting digital health services to large patient groups.

...I think (funder) should consider that on business-led projects they have to be business led, and NHS partners have to really want to do it…you only want NHS partners who see what the project is doing as something that they want to do, so the project is giving them tools or giving them insight. It's not paying them to dabble...

C3 (Midpoint) e-HIT interview 31

Naturally, the efforts required to implement and manage changes are also less complex and costly for smaller organizations and this program allowed them to take risks and test out digital health at scale. Thus, smaller organizations such as consortia of primary care practices were able to be more flexible and more responsive to opportunities presented by dallas communities.

#### Resource Constraints

The health service and other organizations frequently had to contend with major resource constraints during the current period of financial austerity, which affected ability to engage with various initiatives within dallas.

...we’re quite far behind in our IT. We don’t have electronic records as such, so we’re still writing in records. And I think that’s probably half the problem.

Health visitor, Focus Group, 2015

Budget constraints were clearly visible among some partners who struggled with manpower capacity and to provide mobile technology and other equipment for their health care staff to roll out different digital health products and services, which was a major impediment to progressing the digital health ‟consumer” agenda.

### Micro-Level Readiness

Two overarching themes were identified at the micro level: ‟readiness of health professionals” and ‟readiness of the public and patients.”

#### Health Professional Readiness: Workload and Professional Confidence

Workload pressures and lack of capacity was a recurring barrier to incorporating new technologies into everyday working practices and on occasion incentivization had to be used to overcome this.

...it was more difficult at the start but as we have got more patients onto the system, as we have started to be able to say you know this is what the patients are saying, GP practices are warmed to what we are doing and actually become proactive themselves in trying to get their patients onto telehealth. We did have a £600 payment that we would give the GP practices for supplying us with their long term conditions list (...) so it’s just a locally enhanced scheme...Now to be honest they don't even talk about the money now.

C2, Final e-HIT interview 2, health service manager

Concerns around data security, when partnering with private companies, were a barrier to uptake. Some nurses worried that personal information may not be kept confidential and secure or could be sold or shared with commercial companies without the explicit consent or knowledge of patients. Also, given that many digital health initiatives have come and gone over the years and failed to be integrated into routine care in the UK health service, some health professionals were skeptical about the likely longevity of the dallas program and were slow to engage with the technologies it was promoting. In these cases, ‟change fatigue” was evident.

#### Training and Alignment With Professional Roles and Identities

There was a perception by those tasked with driving forward new initiatives that clinicians and others feared digital health as they believed it could be used to disempower and in some cases replace them as care providers.

that’s quite a big initial thought of a lot of different care groups…, that they’d be made redundant by the introduction of technology”

C2 e-HIT interview, representative charity organization

However, the main barrier was not a fear of role replacement, but lack of knowledge and skills in relation to digital health which significantly affected how prepared some were to engage with the different technologies.

...I think the whole system about IT, I am a nurse and that’s what I was trained to do, so before IT came in, we were doing everything on paper, and now things are changing for us, and we’ve never really been given training, we’re only doing it on the job, and we’ve had a new IT system called “x” coming in, that’s created an absolute nightmare for everybody, because we’re not necessarily that skilled in IT processes to be able to do that. So generalized IT training would be good...then tailoring it to the things that we’re doing...

Health visitor, Focus group, April 2015

The pace of technological change was also noted as a problem as clinicians felt unable to keep up to date with new and emerging developments, especially the growth and lack of regulation within the mobile app market.

#### Access to Digital Resources

Even when staff were digitally literate, some found it impossible to drive new digital health services forward because of the technical infrastructure issues reported previously. For example, poor connectivity of mobile devices such as tablet computers and firewalls that blocked their access to internet and social media on NHS sites prevented engagement with new digital health applications and services.

...the key issues were about access to equipment...it was the statutory sector that struggled. ...the security systems that we have on most of our desktops actually stop you getting access to things like (new dallas digital tool). So the browsers were so old that a lot of the material wasn't displaying properly when you were sitting on the public sector end…so it was more to do with the challenges roundabout making sure that staff in the public sector had access to the level of equipment that people take for granted in their day-to-day lives.

C1, implementer interview, June 2015

#### Public Readiness: Digital Literacy and Access

Variation in digital literacy skills caused widespread problems across dallas. The target market ranged from younger consumers—who were more adept, confident, and ready to use digital tools—through to some older adults with little or no previous awareness or understanding of basic IT.

...quite a few of them had no digital knowledge whatsoever, they had no access, they had nobody that was able to show people how to use digital stuff...

Digital champion interview, March 2015

Some consortia had specifically undertaken community-asset based activities to address digital access and inclusion such as setting up digital hubs and creating digital champions to encourage people to get on the Web as many individuals were clearly less digitally “able.” There appeared to be a risk of compounding the ‟inverse care law,” with those from lower socio-economic groups—often most in need of health and social care services—being less able to ‟digitally” access these.

...they give us these and said “Here you are! Here are the tablets,” the first we had seen a tablet was: you took it with water and you put it in your mouth.“

‟House of Memories,” Focus group, March 2015

Despite reported growing use of digital tablets and smartphones among the general population, many people still lacked basic access to such mobile devices. The cost of technology or poor access to computer equipment and free Internet services in local communities prevented many individuals from participating in some of the dallas offerings. To overcome this, one consortium—serving a mixed population including communities in high deprivation—actively sought to develop new routes of access, such as providing education and digital support, as previously reported.

#### Agency of Individuals and Their Perceptions of ‟Consumer” Digital Health Tools

Technologies which required data entry and/or a change in daily routines affected the ‟readiness” of users to adopt these and this proved another challenge for the preventive self-care agenda. The issue of individual ‟agency” arose as people lead busy lives with competing career and family responsibilities, as well as—for some—financial and social demands, which were often prioritized ahead of health.

…People don’t prioritize health, so if you are economically deprived, you prioritize feelings of physical safety and financial safety so you could be worried about paying your rent, keeping debt collectors off the door, anti-social behavior in your neighborhood. If you’re more economically active then other things are a priority, holidays, kids, schooling, housing, mortgage.

C2 implementer interview, June 2015

Some health professionals expressed concern that some mobile interventions were not always necessarily appropriate for their service users—both older and younger—depending upon timing and/ or settings. Equally, while some welcomed new digital solutions, others did not. For example, some individuals were eager to access digital health solutions for elderly parents only to find that the parents themselves were extremely reluctant to permit some form of home ‟monitoring.”

In addition, the increasing multicultural nature of contemporary society presented a barrier when English was the only language available on the digital platforms.

...The only people that I can—hand on heart—say I haven’t offered it to since we started to do this have been a couple of my Polish clients that haven’t spoken English, the vast majority of them speak enough and if they speak and if they can understand me in the booking I will say would you like to access this and often if it’s spoken they can...I have had a couple that have come and there is just not a word of English and for that reason I have chosen not to go there.

Community midwife, Focus group, April 2015

#### Trust in ‟Consumer-facing” Digital Technologies

Trust in digital health security was a persistent issue with some expressing unease about the safety and security of privately held data and whether or not it would be shared with organizations without their explicit knowledge or consent, given recent high profile data breaches.

...I think perception of risk to patient data is a big challenge. People are uncertain about the implications of sharing their data with a system and well it’s difficult to explain the subtleties of the distinction between personally held record which they own the data…while we understand the concept of anonymizing data and who owns consent and everything else those are quite complicated messages to pass to the general public.

C4 final e-HIT interview 4, industry representative

Clinical endorsement and validation was seen as one way to address this and promote uptake and utilization among consumer groups.

## Discussion

Our results show that readiness issues have been ubiquitous across macro, meso, and micro levels and across sectoral boundaries: market, policy, organizational, professional, and consumer. These issues are not insurmountable challenges but their existence does need to be acknowledged and addressed if deployment at scale to the widest population is to be realized.

### Study Strengths and Limitations

We have examined the implementation journey of a national program aiming to deliver digital health at scale across the United Kingdom. We have rigorously collected and analyzed process data from a wide range of stakeholders involved in the implementation, identifying not only potential barriers, but also why these occur and how to address them in future (Table 4). A robust, sociological theory—NPT—underpinned the evaluation as recommended as good practice [32,33]. This could result in inappropriate ‟shoe horning” of data, however, we would argue that we were rigorous in looking for data that also fell outside the framework. Our qualitative data collection was largely limited to those engaging with the dallas program and we did not undertake work with individuals or organizations that were unable or unwilling to engage with the program, which could have provided different perspectives and possibly shed light on digital exclusion or nonparticipation.

Our research was located in 2 countries within the United Kingdom which operate a NHS system where health care is free at the point of access and there are funding constraints which is important to note when considering the implications of this work. In addition, the governments in Scotland and England have a major role in developing and overseeing regulatory and information governance frameworks. Finally, there is a long history of embracing digitalization in health care, for example, all primary care practitioners use electronic medical record systems, which is important to note when comparing the United Kingdom with less digitally advanced countries.

### How Does This Study Fit With the Existing Literature?

Consumer adoption of digital health is seen as a great market opportunity with numerous policy drivers and yet penetration of this large potential market remains relatively poor [37]. Our findings resonate with reports and data from other sources, for example, digital skill and infrastructure deficiencies have been noted by a recent select committee report on digital skills for 2014-2015 [38] that examined challenges for a ‟digital economy” more broadly. The UK government has recognized this issue and vowed to make fast broadband available to every home [39] but our findings suggest this will still leave much to do. Interoperability is a key aim in the United Kingdom as it strives to implement digital standards and achieve system wide interoperability but others have reported interoperability as a barrier to implementation and large scale deployments of mHealth and global health interventions [40,41]. The recent European Union (EU) Task Force on eHealth also acknowledged the need to develop EU-wide standards on interoperability and data sharing [26]. Our whole system view of the digital health ecosystem provides potential explanations, suggesting that interoperability is not a technical issue but rather due to industry inertia and to multiple organizations operating within the health system in the United Kingdom.

Clinical endorsement of digital health products and services including systems for regulation and accreditation of technology and data enabled services is required and has been suggested previously [42], and a recent systematic review of the international literature on barriers and facilitators to patient and public involvement with digital health has suggested this is a key issue [43]. Our work suggests that the health care community would welcome better integration of health records, although persistent challenges are posed by the way current information governance rules are interpreted and enacted. These types of problems need to be addressed if the aim is to share data across sectors. Previous research relating to the use of personal electronic health records has demonstrated less public and professional appetite than anticipated [22,44].

Our work suggests that problems identified in the wider literature on diffusion of innovations such as the importance of structural determinants (such as resources), definite perceived advantages of the innovation, ease of use, good training and support, as well as ability to address perceived risks of new ways [3] of working apply equally to the digital health sphere. Importantly the dallas program has shown that although such issues persist across a range of digital health initiatives more user centered design techniques, intensive engagement, and support of users and incentivization of professionals can help increase interest in digital health.

### Implications for Implementations of Digital Health Technologies at Scale

The dallas program highlighted challenges but also potential solutions to the large scale implementation of digital health, for example, through the development of information governance recommendations for health care organizations [45] and the use of digital champions to address skill deficiencies. Our findings lead us to a set of actionable recommendations for future work and for increasing readiness for digital health at scale (Table 4).

**Table 4:** Recommendations for future implementation work in digital health.

Recommendation no.	Recommendation
Recommendation 1	Further commitment and investment in both national and local infrastructure will be required if digital health care is to become normalized.
Recommendation 2	Guidance relating to ownership and control of personal health data and data privacy regulations are required to mitigate current uncertainty in the digital health arena.
Recommendation 3	Brand trust and confidence is crucial. Accreditation and official endorsement of products and services is an important determinant of future successful deployment of digital health services as is peer recommendation for consumer wellness products. Clear systems to facilitate trust and confidence need to be put in place.
Recommendation 4	Technical and service interoperability needs to be prioritized and, if necessary, incentivized to ensure the scaling up of digital health care across systems and sectors.
Recommendation 5	Future digital health services need to be more accessible by those who are currently socially or economically excluded including those whose first language is not English, and those with sensory, physical, or cognitive impairments.
Recommendation 6	There is a need to invest in further awareness raising, upskilling of consumers and more affordable and accessible technologies if the true potential of digital health and wellbeing technologies are to be fully realized and the concept of professional and lay champions to promote technologies and services merit support.
Recommendation 7	More extensive and intensive public engagement and debate on the subject of the risks versus benefits of digital health needs to be undertaken to address concerns around security and safety of digital health and wellness products and services.
Recommendation 8	Greater emphasis needs to be placed on both upskilling and also ensuring the next generation of health professionals are more ‟digitally” able. Digital health care needs to be a feature of undergraduate health professional training.
Recommendation 9	Guidance is required to shape and support a market that spans consumer wellness and statutory health services. Consideration must be given to future funding models, procurement, and the potential for hybrid data, including sharing, storage, and management models that permit digital health apps and services to be taken up and used via consumer markets and/or statutory channels.
Recommendation 10	There is a need to promote health care stability and a culture of long term planning. Instability and constant change can be a deterrent to investment and hinders implementation in the digital health sphere.

### Conclusions

Although there is much rhetoric about the consumer push for digital health, our research raises some outstanding issues relating to the readiness for digital health that need attention. We provide a set of 10 key recommendations that aim to tackle these issues. If addressed, these recommendations will promote the right market and environment to permit the routine deployment and true scaling-up of digital health and wellness technologies and services.

## Appendix

Multimedia Appendix 1 Overview of dallas communities.

Multimedia Appendix 2 The 4 normalization process theory (NTP) constructs.

Multimedia Appendix 3 Qualitative tables with additional study participants’ quotes.

## Abbreviations

dallas: Delivering Assisted Living Lifestyles at Scale
NPT: normalization process theory
