# Supplementary material for: Readiness for Delivering Digital Health at Scale: Lessons From a Longitudinal Qualitative Evaluation of a National Digital Health Innovation Program in the United Kingdom
Source: J Med Internet Res. 2017 Feb 16;19(2):e42. doi: 10.2196/jmir.6900 (PMC5334516; doi:10.2196/jmir.6900)
Supplement: Multimedia Appendix 1 [file jmir_v19i2e42_app1.pdf]

Readiness for Delivering Digital Health at Scale: Lessons From a Longitudinal Qualitative Evaluation of a National Digital Health Innovation Program in the United Kingdom

Lennon, Bouamrane et al. 2016

APPENDIX 1: Overview of dallas Communities

1 (a) i-Focus (iF)

Overview: i-Focus is a community with a focus on interoperability. They also developed prototypes of both statutory and consumer targeted reassurance services to promote and encourage a more proactive self-care or self-management approach. A key output of i-focus is the national membership based digital health interoperability community called DHACA.

Location: National

Partner Organisations: Lead: *Industry* - Advanced Digital Institute (ADI)

Industry Partners: Microsoft, Healthcare Gateway, KPMG, Tunstall, Tynetec, TheAlloy, The Future Customer, Canary Care, Lumi Mobile, Sitekit. *NHS:* Innov8. *State and Government Services*: Health Design & Technology Institute, CarersUK,

**Services and Products**	**Description**
AroundMe<br><br>*Canary Care*	An informal reassurance care platform that uses a range of connected home sensor technologies to support older or vulnerable people in their home. Learnings from prototype development being leveraged to support the roll out of CanaryCare.
AboutMe	An e-learning service for carers developed with Carers UK.
PainSense	Mobile app for promoting self-management of chronic pain. Developed by ADI and NHS England leveraging learnings and architectures from dallas.
Self-Care Hub	Online portal acting as digital advisor and front-door to healthcare services for people with chronic illness. Deployed in Kirklees in 2015.
DHACA	National interoperability membership based community providing a source for the learnings from the programme and support to exploitation across the sector.

1 (b) **Living it Up (LiU)**	

Overview: Living it Up is a digitally enabled community of opportunities (aimed primarily at people over the age of 50 and people living with long term health conditions) to support better health, wellbeing and active lifestyles in Scotland. A key activity was co-design in the community and a key output was a web based portal signposting people to health and wellness services across Scotland.

Location: Scotland

Partner Organisations: *NHS* - NHS 24, NHS Lothian, NHS Western Isles, NHS Forth Valley, NHS Grampian, NHS Highland. *Voluntary Sector* - Carers Scotland, Alliance Scotland

Industry - O2, Vodafone, Highlands & Islands Enterprise, Atos, Philips, Intersystems, STV, STV Health Centre, Maverick TV, Ernst & Young, Scottish Enterprise, Illuminadigital Ltd, Intrelate, Sitekit, Looking Local. *Academia* – Glasgow School of Art. *State and Government Services* - Scottish Government, Kirklees Council, The Highland Council, Argyll & Bute Council, Moray Council, West Lothian council, East Lothian Council, Edinburgh Council, Falkirk Council, Stirling Council, Clackmannanshire Council, Midlothian Council.

**Services and Products**	**Description**
Liu Portal	A web based portal acting as a single access points to the range of services offered by LiU **(**https://portal.livingitup.org.uk/)
Shine	An online profiling service to identify individuals' skills and expertise along with encouraging members to help others to share their own skills and "talents" in their communities. (https://shine.livingitup.org.uk/)
Connect	A means for people to remain 'connected' with their friends, family and care-givers via Skype or Cisco Jabber Client video conferencing (VC) suite. (https://portal.livingitup.org.uk/connect)
Discover	An online portal with access to information on a range of health and social care products and services available in local communities. (https://shine.livingitup.org.uk/discover/Searching).
Flourish	Suite of interactive tools to support self-management of long term conditions ('experience guides', NHS resources, a home monitoring service and a text messaging service. (https://flourish.livingitup.org.uk)
Get Active	A service developed in collaboration with Storm Health which aims to promote and support healthy activity for targeted individuals.

1 (c) More Independent (Mi)	more independent

Overview: More Independent (Mi) is a Liverpool based partnership led by Liverpool (NHS) Clinical Commissioning Group. Mi aimed to enable citizens take control of their health, well-being and lifestyle. Key outputs include the role of both lay champions and clinical champions in increasing uptake and use of digital health and self-management.

Location: Liverpool

Partner Organisations: NHS Liverpool Commissioning Group (CCG), NHS Liverpool Community Health Trust, Person Shaped Support, NHS Informatics Merseyside, Riverside (*social housing*). *Voluntary*: Hft *Industry*: Sitekit, Philips, Tunstall.

Services and Products	Description
Mi Awareness	People invited to engage in community events, and join both virtual and physical community interest groups.
Mi Health and Care Technology	Scaling the deployment of health and care technology across to enable more people to live healthier independent lives and give families (inc carers) peace of mind. www.moreindependent.co.uk/wp-content/uploads/Mi-Telehealth-Report.pdf
Mi Plans	Online tools (i.e. personal health records) to increase people's ability to manage life-enhancing plans to support self-care.
Mi Retail	An initiative is to improve the visibility and availability of Digital Technologies to local communities by engaging with the retail sector in Liverpool. www.moreindependent.co.uk/wp-content/uploads/Mi-Retail-Report.pdf
Mi LET helpline	A telephone hotline to provide advice on health and wellbeing technologies and services.
Mi Champions	Active volunteer citizens who work in local areas to promote and advise people of the telehealth and telecare services available to them. www.moreindependent.co.uk/news/mi-community-champions/
Mi Digital Inclusion	Initiative to reduce digital exclusion by creating digital hubs in areas of low digital access. www.publictechnology.net/articles/features/delivering-health-care-digitally-excluded
	A (real and virtual) Demonstrator Smart House equipped with a wide variety of

Mi Smart House	technology to make life easier.<br><br>www.moreindependent.co.uk/news/come-and-visit-the-mi-smarthouse/
Mi 'House of Memories' App	App designed to support people living with dementia and their families. www.moreindependent.co.uk/news/mi-house-of-memories/
Mi Health Innovation	Supporting local business to innovate, opening up new forms of finance to support growth, up-skilling the workforce and digitally including citizens.

1 (d) Year Zero (YZ)

Overview: Year Zero is an industry led consortia bringing together expertise in healthcare, design, media and technology to develop user-centred tools that will deliver new digital healthcare services and enable citizens to take greater control of their own health and wellbeing. A central objective was to test the utility of new services designed around personal health records and digital interactions between citizens and service providers.

Location: West Midlands, Liverpool, Grampian and London

Partner Organisations: *Media Industry Lead*: Digital Life Sciences.

NHS and Social Care: Locally Healthy (formerly NHS Local), Liverpool Community Health Trust, South Warwickshire Foundation Trust, Moray Community Health and Social Care Partnership, The Rotherham Foundation Trust, the Vitality Partnership.

Industry: Sitekit, Kameleon, Maverick, Microsoft.

**Services and Products**	**Description**
eRedBook (eRB)	A digital version of the printed Redbook (a Personal Child Health Record (PCHR) that logs a child's healthcare information such as developmental growth and milestones, vaccinations, and other information from birth. http://www.eredbook.org.uk/
Health United Birmingham HuB	The roll-out of remote live consultation and extending out of hours services with the Vitality Partnership in Birmingham. www.digitallifesciences.co.uk/digital-life-sciences-helps-improve-patient-access-to-gps/
A Better Plan (ABP)	A care planning application that is designed to help healthcare professionals and patients to collaboratively consider and set a range of health, wellbeing and personal

Vitality Plan	goals. https://www.abetterplan.co.uk/
Good Neighbours	A social networking application that allows users to create circles of support with family members, friends and carers and to share and allocate tasks and diaries within an online social support network. www.mygoodneighbours.co.uk/
No Delays	An application that allows GPs to prescribe a digital postcard (a personalised package of short videos that explains their condition and informs them about available local services) by email to the patient. www.nodelays.co.uk
uMotif	A mobile and web application designed to support self-management for patients with Type 1 and 2 diabetes. https://www.umotif.com/
EDaybook	A prototype application for staff (Carers) working in Domiciliary Care to support management of care plans and to facilitate improved communication between Carers.<br><br>http://www.digitallifesciences.co.uk/ive-got-better-plan/
