# Supplementary material for: Readiness for Delivering Digital Health at Scale: Lessons From a Longitudinal Qualitative Evaluation of a National Digital Health Innovation Program in the United Kingdom
Source: J Med Internet Res. 2017 Feb 16;19(2):e42. doi: 10.2196/jmir.6900 (PMC5334516; doi:10.2196/jmir.6900)
Supplement: Multimedia Appendix 2 [file jmir_v19i2e42_app2.pdf]

Readiness for Delivering Digital Health at Scale: Lessons From a Longitudinal Qualitative Evaluation of a National Digital Health Innovation Program in the United Kingdom

Lennon, Bouamrane et al. 2016

APPENDIX 2: The four Normalisation Process Theory (NTP) constructs

NPT Construct	Themes Addressed by Construct
**Coherence**	Can people make sense of the new way of working and understand the purpose of the service innovation?<br><br>Can people see how the service innovation differs from their current work practices?
**Cognitive Participation**	Do people buy into the new ways of working and can they sustain the new way(s) of working?<br><br>Can they persuade others to join in?
**Collective Action**	How easy it to work in the new way?<br><br>What resources/support/training are needed?<br><br>Are people confident about the new way of working?<br><br>How does it affect roles/responsibilities?
**Reflexive Monitoring**	What do people think of the new way of working?<br><br>Can people modify the new way of working to better fit their needs?
