# Supplementary material for: Readiness for Delivering Digital Health at Scale: Lessons From a Longitudinal Qualitative Evaluation of a National Digital Health Innovation Program in the United Kingdom
Source: J Med Internet Res. 2017 Feb 16;19(2):e42. doi: 10.2196/jmir.6900 (PMC5334516; doi:10.2196/jmir.6900)
Supplement: Multimedia Appendix 3 [file jmir_v19i2e42_app3.pdf]

Readiness for Delivering Digital Health at Scale: Lessons From a Longitudinal Qualitative Evaluation of a National Digital Health Innovation Program in the United Kingdom

Lennon, Bouamrane et al. 2016

APPENDIX 3: Qualitative Tables with Additional Study Participants' Quotes

Illustrative data excerpts relating to MACRO readiness issues

**MACRO Level**
Market Readiness
**Interoperability**
"…And I think there is a, sort of, an element of that maybe we could work a bit more together, but the other thing I've learned is where do I spend my time? Do I spend my time in a meeting in [*Organisation Name*] discussing data integration with another company when I've got…., only in the tens of thousands users worldwide, or do I try and get into the 20, 30, 40,000 users worldwide and then worry about it?" **[DHACA Interviewee No. 2, 2015]**
"I used to work for a technology company, I know how it works. You know, you do something first and it's very high risk, very painful and very expensive. If you replicate that, it gets cheaper, lower risk and you make more profit, but you don't tell Company B that you cracked all this and you took all the risk last time around." **[Final e-Hit C4 Interview 1]**
"Interoperability is not primarily an intellectual technical challenge, it is more about finding grounds of co-operation between competing commercial interests and professional interests in individuals…" **[Final eHit C4 Interview 3]**
"there remain significant incompatibilities between the different technical and service architectures within the dallas communities, which is probably inevitable given, first of all, that each community has got a very different context and a different legacy that it needs to manage, and also that the i-focus work started concurrently with the dallas communities, rather than before it." **[Midpoint e-HIT C2 Interview 2]**

**Risk & Liability**
"It's a young person's market, or it's a 'worried well' market. So the people who buy fit-bits, and pedometers, and things, are the 'worried well', or they're gym bunnies, who are, like, competing to see… run the fastest and the most in a week. And that's great, [..] because ***they*** are responsible for the outcome of that monitoring. If you're monitoring a heart condition, you want someone at the end of that. You don't just want a fit-bit, you want a Triage Nurse, because there is an impact. So I think I'm… I'm not entirely convinced that there is a Consumer Market in these technologies, actually…. I think there's *a hybrid market,* maybe, in these technologies, but I think it's going to be… And I think we might be a way off, you know, finding it, but I think it is…really complex. **[DHACA Interviewee No. 9, 2015]**
"We are bringing in a person health file/ record which means that the person who owns the record can share the record and that starts to blend into the integrated health and care agenda for xxxx so again from a strategic perspective I think Community X has got the foundation levels in place to build something that can be really powerful for scaling up." **[Final e-HIT C1 Interview 1]**
"…there are some real threats just in terms of basic data security and public understanding that if not addressed in a proportionate way could actually create some bad stories which are going to put the whole market back so I think there is some real problems there, some of them which are political, some of which are cultural and some of which certainly the data security pieces are technical." **[Final e-HIT C4 Interview 3]**
"I mean, the setbacks we've had [Company X] were looking to enter this market. It was slow for them, and again I can understand why ….because ……when you're entering a new market, it's critical that you've got brand alignment…… And when we started working with [Company X], again, that was the conversations we were having with them: well, how does this align ….., because obviously the brand value is critical, and if you're entering this market and it. But eventually …the brand alignment that we were starting to evolve with them, and we were... we were making good progress with them. Unfortunately, we got to the point in February where bills... energy bills were starting to hit doormats and they got called in front of the government select committee and got beat up, as they do every year for high energy bills, and they said, look, it's the wrong time for us to look to enter this market because we're getting beat up over here over our core offering, and we also need to focus on that. I mean, they'd actually set up... you know, they'd appointed a product manager, we'd got a product management team, you know, all employed and working on it with us, so they had to pull back at that point." **[Midpoint e-HIT C4 Interview 1]**

"… called, in the event that your Mum falls over. So if she falls over, there is a Monitoring Service that calls these six numbers. So it is sold, your pendant Alarm is sold with a Monitoring Service attached to it, and that's why it's sold through [Third sector organisation 1] and that's why it's sold through [Third sector organisation 2], because they are trusted brands, and so you trust the back end service. And if your pendant's provided by your Sheltered Housing Accommodation, by your Housing Association, or by your Local Authority, you trust the Monitoring service. So when we talk about community equipment, community alarms, we are always talking about managed service; we're not talking about kit. So in this field, people keep trying to talk about a consumer market for kit. There isn't a consumer market for kit because they're not selling kit. They're selling a service, so, you know, all the lovely X remote consultation stuff has to be managed by someone.<br>**[DHACA Interview 9 2015].**
"…there is a particular approach to the way data is managed and controlled between the internet and the professional systems that are done in a particular way will meet the information governance and security concerns of the health system as it currently stands while allowing innovation around personally generated data that can be used to provide support for self-management for that individual but can also in a controlled way be used to inform professional decisions so I think that considering where we were 3 years ago I think that the conversations are much more sophisticated now and better informed and are much, have greatly matured during the course of dallas."<br>**[Final e-HIT C4 interview 3]**

**Clinical Endorsement of Digital Health Products/Services**
"….what our experience brought us to realise is that people will only use a kind of Personal Health Journal and plan around serious conditions or long term condition*s if it's something that they can engage with their Clinicians on and* that was kind of a back-to-front approach. You're not really going to persuade people to go out and buy it as a consumer product if it's not something that their Clinicians will engage with them on and look at and share the information that they've been collecting.." – **[C3, Implementer Interview, June 2015]**
"Clinical organisation X want to control pace of change, pace of implementation. And, although the initiative is owned by patients or the parents , the natural corollary of that, which is yes, we want to build something that works for the health service, but the front of it …is not any different to users, and the idea of releasing that to the public, rather than keeping a tight control over it, they just can't countenance. They don't see the benefit, which is glaringly obvious, they don't see the risks and they don't like the pace of change. …" **[Midpoint e-HIT C3 Interview 3]**
"We can't keep up with it, physically we can't keep up. Anyone can put an App on and we can't physically keep up with what's a good app and what's a bad app. " **[Focus group, April 2015, user/health professional]**
"….it's almost like …create a different sort of structure which will enable us to market it almost to Primary Care and GPs and Community Nurses? Also, we're getting feedback from some GPs that we're consulting with to attach it to campaigns like flu campaigns, drug campaigns, you know, Diabetes week, you know, to go down that route… as well where we're actually linking it in… So we've produced flyers, we've got a Junior Doctor who is going to basically promote initiative X directly to these patients as they sit in the waiting room and, you know, go in and out for their flu jabs." **[Midpoint e-HIT C3 Interview 1]**

Complexity of access to UK market
"…for us to have gone to the CCG with that idea you know the amount of red tape from the NHS and the amount of committees and things you would have had to go through and finance committees, procurement committee, it would have been very difficult you know to even know who would be the right person to speak to so as I said one of the best things about x in my opinion is that it's kind of you know opened opportunities for people to get round the table and have real discussions about how they can make a real difference and you know that's been a really positive part of the programme." **[Final C2 e-HIT interview 1, social care manager]**
"So the barriers were around brand alignment, timing, market receptivity, the fact that there wasn't a market that you could easily see at the minute to say, yes, I'm going to jump straight into this. So ...new market entry, was the biggest barrier." **[Midpoint e-HIT C4 Interview 3]**

Policy/Legislation Readiness
**National Policies and Legislation**
" … There is a real problem in that all ….health data is in a Vault that's owned by the NHS. You can't, *at the moment,* view it and when you can view it, i.e., when we get our online patient records, it will be a view which is not in a form that can be used by technology *outside of* the NHS in any real useful way. I think the biggest issue is **Information Governance** and letting people take ownership of their own data and their own risk appetite, and until that happens all we are doing is allowing the market to develop outside of the true record. ………The barrier is the NHS and their vault mentality on our data." **[DHACA Interview 3, 2015]**
"We're working on e-mail Referrals for Carers' assessments which, if we can get e-mail Referrals for Carers' assessments then that opens up a whole new world. The reason for the complication is you've got data going from the GP Practice through the Local Authority into a Third-Sector organisation and at the moment there isn't that ability for [company X] to talk directly to the Third-Sector organisation." **[C2(Midpoint), e-HIT Interview 1]**
"When we are sitting at work, we are not allowed to do that, we can't access 'Twitter' when we are work but we can put it on our phone, onto to our devices and obviously you do that as ***an individual*** rather than ***a professional*** but quite a lot of the online engagement that we are trying to do as a digital[….] they are using Social Networking….and we can't do *that so* ***the Information Governance challenges have been seriously significant for us*** and I think that's going to be true for any kind of Digital Service as we forward and it's just something that we are going to have to take in our stride and come up with solutions…" **[C1 Implementer Interviews, June 2015]**

"…you know the Benefits Reform has been a great carrot or stick whichever way you look at it to push people into doing that, you'll hear stories quite often from Digital Champions, people coming in on a Friday afternoon because they are going to get sanctioned if they don't do this online form….." **[Digital Champion Interview, March 2015]**
"We've got a share so the patient can email their own information off to their friends and family if they want to so you start to promote this use of, well I want to share my data with whoever and that sort of starts to bring a bit of momentum into the whole system and say well you know why, it's my data why can't I use it?" **[Final e-HIT C4 Interview 2]**
"So the interesting thing about initiative Y is the Health Service.., the NHS in England talk about it as your record, but it isn't. The record that they care about is all the data that they've torn off to put in their system." **[Midpoint e-HIT C3 Interview 3]**
"I think probably the biggest worry with people whenever you put in your own information into something that is online is security and how safe is my information. So I think that's a big barrier potentially that we will have to get over with a lot of people about, reassurance that their information is safe." **[Midpoint e-HIT C3Interview 2]**
I think it's been really interesting the last couple of years, just seeing things like the iPads, the issues that come up, things people want to talk about, things they find hard to accept about putting an iPad in, it doesn't fit with your Windows infrastructure, it empowers a user to a scary degree where maybe they're going to break all your security mechanisms and use Drop Box and all these terrible things. Yet actually, does that happen?? or has it just facilitated a different way of working. I think it's the latter. **[Midpoint e-HIT C4 Interview 3]**

**Infrastructure**
"…I think there's probably a wee bit of, not scepticism, probably more concern as to that all sounds great, but do we have the infrastructure here to be able to allow us to do those things…?." **[C1,(Midpoint) e-HIT Interview 8]**
" Area x did phenomenally well given the lack of, the poor connectivity in the region you know the poor WIFI and even when we 3G enabled their tablets poor 3G signals you know so they did very well, it was a lot of hard slog I think of going around child centres and signing people up. " **[C3 Final e-HIT Interview 1]**
"Access to fast reliable IT is an issue. Pervasive daily pressures impede adoption" **[No delays Free Text Survey Data]**
"People have to know about the technology and need to be able to access it, because what we found is there are big portions of the community out there that can afford it but they can't access it." **[Midpoint C2 Interview 5]**
"Yes, the other significant area is Mobile, very challenging, mobile coverage is frail, in terms of it comes and goes, but where it does exist, and in many places it just doesn't exist." **[Midpoint e-HIT C 1 Interview 8]**
"I think there are other challenges which we're taking care to do with the Telecommunications infrastructure, you know, that's required for this but you know, x are investing £150 million in upgrading the most challenging parts of the infrastructure to bring greater backwall capacity to all x, and to bring high-speed broadband; in reality being some of the solutions required don't really need huge bandwidths." **[Midpoint e-HIT C1 Interview 9]**

Illustrative data excerpts relating to MESO readiness issues

MESO Level
Industry Readiness
**Lack of Market Coherence**
"You know if there is one thing that we can give the evaluation processes is what not to do on the retailing because you know we kind of, you know we tried to get it in a shop, we have spoken to some of the retailers and you know we tried selling stuff in the x shop and you know we have tried a whole range of things where it's had some success but it's had limited success." **[Final e-HIT C2 Interview 1]**<br><br>"….so first of all I think the User Experience is key so …start with the language of the consumer and the language of the value proposition and all of that, the people who are having to sell to consumers - who are parting with their money - rather than looking at the language of Local Authorities and Health Sector which is you know all about sort of Cost Avoidance and *Tier this* and *Tier that*…. it's understanding what do people really value so what we found was that they value reassurance, they don't want alerts, they want reassurance…….." **[C4 Implementer Interview, June 2015]**<br><br>"…….. and even the User Experience because, obviously, a 75 Year old that has never used a computer will need something slightly different from the 52 Year old that works in an office and uses a computer every day you know ?? So I think that has been quite a, quite a challenge to actually get the tone and the language and the User Experience right for that broad target audience…." **[C1 Implementer Interview, June 2015]**<br><br>"I think the big challenge that we've got is that if you look at a pathway, it's difficult, a health pathway, it's difficult to identify the practitioner on that pathway who takes responsibility for setting outcomes and achieving outcomes, it's in health,….it's far further behind social work practice in that space." **[Final e-HIT C2 Interview 3]**<br><br>"I think what has worked well is actually bringing a consortium together that has allowed everybody's mind to be opened to new ideas, new ways of working, new methodologies….. we have definitely moved all of that on, agile development, we've upskilled technical partners, co-design, community engagement we've upskilled traditional partners and actually thinking this is the future of engagement and working with the general public in designing new services." **[Final e-HIT C1 Interview 1]**

"…in certain areas there has been resistance to change from certain individuals I wouldn't say people operate as organisations so it very much depends on where your champions are and to what extent they are influential and people are busy they've got hundreds and thousands of things that they are trying to do at the same time particularly in environment of significant change but I would say that where we are engaging there are good local champions who are taking it forward, undoubtedly they will have resistance as any significant change programme has resistance but they seem able to work their way through that and continue to get the engagement and traction that they need." **[Final e-HIT C1 Interview 2]**
**Collaboration, Competition, and Codesign**
...It's a sort of codesign, and what happens is people take a long time to make up their mind which compresses time for technical partners (...) for some people working in a collaborative nature with technical partners is a new environment so they are cautious and wary of telling all their secrets in case people run away with them so I think there is a protective defensive mode. **[C1 (Midpoint), e-HIT interview]**<br><br>"........so the beauty of the dallas project is its collaborative aspect which allows us to be more innovative." **[C2 midpoint ehit interview 4 Industry]**

NHS (Organisational) Readiness
**ICT Infrastructure**
"Well things like legacy systems, fire walls, when we are adopting new technologies, eHealth capacity, e-health priorities within the internal infrastructure is stretched." **[C1, Final e-Hit Interview 1]**
"Our IM&T team X have been really good because they have helped tailor the EMIS system for us…so we have our own caseload now within [Company X] where we can track the patient journey and that's what informatics X have done for us. We are also looking of a way of reducing down the amount of things we need to double key in." **[Final eHIT C2 Interview 2]**
"…but in their (the trust's) mind, the cost of implementing x, because when we started the project, their Tablet, it was presaged on the idea that their Tablet X working would be ready. That would be in place nine months into the project, by the time we might be running to catch up. Instead of which, two years in, it hasn't even started." **[Midpoint e-HIT C 3 Interview 3]**
**Discontinuity and Organisational Culture**
"….there is *huge* change going on in the public sector just now, both…. and kind of Health and Social Care landscape and lots of re-structuring, changes in staffing so….. So actually, it's then difficult to keep people focussed on what they have got to do when they have got a wide range of things that they are looking at all the time and there is so many changes happening..". **[C1 Implementer Interviews, June 2015]**
"… Because again there was *that lack of Senior Buy-In* and an ability to push things through and also [NHS region name] for the last year or more was going through a major period of structural change an upheaval as well. So that's what you are dealing with when you are dealing with the NHS organisations they are trying to do their day to-day business as well as dealing with, well what's the word, investigations and you know changes in structure, changes in strategy all of that going on around them so it's a moveable feast…." **[C3 Implementer interviews, June 2015]**
"Oh yes a lot of the time at those Senior levels of an Organisation the outcomes are known and the inputs are known but what they don't understand *is how those inputs have ended up in the outcomes*…….but it's staff and those people who have to implement it that may need to move *in a slightly different direction in order to achieve what we need to achieve.* ***And doing that is such a complicated beast***, and I think that will be such a key skill and such a key thing to understand because it's such a complex system that you cannot, in my view, simply commission e-Health technology – it can't be done – because there are too many stakeholders who could block, misunderstand or simply just not want to get involved…." **[C2(Midpoint) e-HIT Interview 5]**

"… Because, if it fitted in with *their* strategy, and they were getting a product or new things to try or things, the resource was the project. Well, I do think it's something that TSB should consider that, actually that one of the rules on business-led projects….is they have to be business led, ***and the NHS partners have to really want to do it***, and the amount of money that the TSB can contribute isn't enough to make a difference. ….So, the real criteria should be you only want the NHS partners who see what the project is doing, as absolutely, ***as something that they want to do***, so the project is giving them tools or giving them insight. It's not paying them to dabble…." **[C3 Midpoint e-HIT Interview 3]**
"……a very switched on GP practice who were keen to change their business ……. they were taking a very business -like approach to how their practice should operate and were looking at models for greater efficiencies and dealing with larger numbers. They had a 60,000 strong patient base and growing because they are a consortium of GP practices so they have been acquiring other GP practices as they go and increasing in size all the time and they were very quick to see the benefits of digital engagement…." **[C3 Implementer Interview, June 2015]**
"We tried that as well but we kind of didn't know how to navigate it, this comes back to my issue of the due diligence of the partners, you expect that the partner organisation knows those people and will have those conversations to enable it to happen in their organisation. What became apparent with Organisation X is that wasn't happening, there was nobody senior enough in Organisation X to open those doors and have those conversations and the most senior person from the E health side that we were dealing with was really obstructive to going forward." **[Final e-HIT C3Inteview 1]**
Well, the one I always say to people is don't underestimate the time it takes to do things…I've got a corporate background and I've been involved in big change programmes so it wasn't really a shock to me, it was a reinforcement of something I already knew after 35 years working in a big corporate organisation, don't underestimate the time it takes to create significant change. Again, similarly, it's more a reinforcement, what I was talking earlier about, you need to create space to change, and that space is created by the provision of appropriate resources, which could be time, it could be money, it could be indirectly money, so extra people giving you that extra capacity, to do something. The other one is the alignment of all stakeholders to that change and to create that motivation to change. **[Final e-HIT C4 Interview 1]**

Resources
“So we’ve really gone at, you know we have gone at pace and we’ve done that because we have had really strong support from the CCG and CCG are the guys that can help corral the GPs you know so that’s been, that’s been key, sharing agreements is the other thing to make sure you have in place you know making, it was really good that we had the LMCs to be able to get the patients records, the [Company X] records to help manage them when they are on the telehealth so having those things in place makes it a lot easier.” **[Final e-HIT C2 Interview 2]**
“I would say strategic lead engagement has been excellent, we’ve got very good links into senior management levels within the local partnership areas from the industry partner perspective it's very much that senior management level that champions any issues or changes that need to be made so no I think engagement continues to be very good.” **[Final e-HIT C1 Interview 2]**
“..one of our partners were worried that we wouldn’t have the product X ready in time for their digital roll out a year into the project, they hadn’t even started their digital roll out they were so far behind their own organisational strategy, turns out they didn’t have tablets, they didn’t have i-pads, they didn’t have connectivity, they didn’t have you know loads of things but you are now in bed with these partners and you are trying to make it work you know so I think a huge amount of effort was spent trying to make these artificial relationships work.” **[Final e-HIT C3 Interview 1]**
“And some of our work, even for example, one of our big projects just now is around mental health, and we probably will not be in a position to actually decommission our current services, and re-commission the new services, until we actually have capital funding from X. So, we’re probably talking about that being a couple of years away. So with the time...the change fund was available to do that, so it...you know, sometimes the timing for these things needs to be a bit more fluid than, you have these years to do it. That can make it really difficult. So, yes, that is going to be a real pressure.” **[Midpoint e-HIT C1 Interview 10]** “...we’re quite far behind in our IT. We don’t have electronic records as such, so we’re still writing in records. And I think that’s probably half the problem”. **[Health visitor, Focus Group, 2015]**

Illustrative data excerpts relating to MICRO readiness issues

**MICRO Level**
Health Professional Readiness
**Workload & Professional Confidence**
” ……I guess my main point that I’m trying to make is that it was more difficult at the start but as we have got more patients onto the system, as we have started to be able to say you know this is what the patients are saying, you know, the GP practices are warmed to what we are doing and are actually you know become proactive themselves in trying to get their patients onto telehealth. We did have, and it's probably fair to add this in, we did have a £600 payment that we would give the GP practices for supplying us with their long term conditions list…..So it's just a locally enhanced scheme……Now to be honest they don’t even talk about the money now.” **[C2, Final e-HIT interview 2, health service manager]**
“I think the key lessons are probably, it's I think the big thing has been never assume because you know as I mentioned one of the biggest, or most surprising things I found was that a number of GPs had no idea what telehealth or telecare was.” **[C2 Final e-HIT interview 1]**
“Because you know again it's a short time funding opportunity even although it was significant funding over that kind of [………..] period and you know again traditionally when you work in the public sector you see lots of things come and go and you do get a bit nervous about engaging to rigorously all about it and then you find that it' not there in six months’ time and you’ve been sign posting to it so there is a bit of that about in terms of its life span which we need to address” – **[C1, implementer interview, June 2015]**
“…you’ve got a group of champion health visitors who think yeah I’m prepared to double my workload, I’m prepared to fill in the paper version and duplicate some of those answers into the initiative because I can see that this is going to be a nice way to work in the future and if we can just go on to tablets only and the parent has a copy on their phone or their pc or whatever that’s great they bought into it, the supporters of it bought into it well but if you are then sort of saying to them a year, 18 months on we are no further to having this integrated into our work processes at x they start to lose interest you know they start to see this as just an ongoing exercise, no end in sight and it's very hard to keep that motivation up so that’s the conflict between a funded project with a set of aims and objectives and how you integrate that into the overall organisational structure.” **[Final e-HIT C3Interview 1]**

"I think one of the key challenges and i think you will find this across the whole of the UK is clinical resistance in a way because they are so busy that they don't have time to change and they don't, and also chicken and egg, because they don't have time to change they don't want to try it because you don't have the evidence but you can't get the evidence unless they try it." **[Final e-HIT C1 Interview 1]**
"…this is just my impression from the conversations that I've had, but that there is... there is certainly a sense among GPs that they are beleaguered and that they're being put under enormous pressure and they don't have any time to change, and that they're being blamed and criticised and all this, so I think there's... they're in a very defensive mind-set. I think they are very suspicious of initiatives... any initiatives which will ask them to do anything differently." **[Midpoint e-HIT C4 Interview 2]**
"I think our challenge is actually on the clinical side and the mind-set change that has to happen, that people could actually potentially self-manage and give them that ownership over that. I think that's one of the biggest challenges. We're still ...as clinical staff protective over our patients, thinking… and risk-averse, I suppose, thinking that, actually, they don't have the ability to look after themselves; and we have that traditional 1940s methodology, that: don't worry, we'll fix you…than actually trying to empower them with the relevant tools to help themselves. So I think that's a massive barrier….Also, I think we often find it difficult as a service provider to think about innovation and to think about what would really add value, because they're so entrenched in the process of filling out forms, and then that form goes there, and then what happens next? They actually can't think that it could be done differently." **[Midpoint e-HIT C1Interview 11]**
**Training & Alignment with Professional Role/ Identity**
"…How do we work with Health and Care practitioners to help them understand this agenda of self-care and technology *isn't trying to disempower them,* isn't trying to take their jobs from them, it's an enhancement to what they do, it's an enhancement *which will increase service capacity*. So again this isn't a cost cutting exercise, this is an exercise to help you do more for the people that you work with and actually it's something that can enhance your job role." **[C2, Implementer interview, June 2015]**
"….I think the whole system about IT, I feel first and foremost I am a Nurse and that's what I was trained to do, so before IT came in, we were doing everything on paper, and now things are changing for us, and we've never really been given training, we're only doing it on the job, and we've had a new IT system called 'Lorenzo' coming in, that's created an absolute nightmare for everybody, because we're not necessarily that skilled in IT processes to be able to do that. So generalised IT training would be good, as well as then tailoring it to the things that we're doing. …" **[Focus Group, April 2015, Health visitor]**

"I don't use it as a routine part of my work. I would like to and can see its value but the training and demonstration of how to use it has been dreadful" **[No delays – free text qualitative data]**<br><br>"……..that's quite a big initial thought of a lot of different care groups……., that they'd be made redundant by the introduction of technology"<br><br>**[C2 - e-HIT Interview, - representative charity organisation]**
**Provision of Suitable resources**
"..the key issues were about access to equipment and I'm trying to think of the right, political terminology for all of this but it was the Statutory sector that struggled more. That some of the access they had to things like iPads …. the security systems that we have on most of our desktops, our PCs. Actually stop you getting access to things like [Name of new dallas digital ool/service]. So what we were having to do was to go in Browsers, the Browsers were so old fashioned that actually a lot of the… material wasn't displaying properly when you were sitting on the Public Sector end …..so it was more to do with the challenges roundabout making sure that staff working in the Public Sector had access to the level of equipment that people take for granted in their day to day lives and accessing all the time." **[C1 implementer interview, June 2015]**
We have been unable to get WIFI in our practice, no one has responded to me contacting the email I had to discuss my difficulties ….. I seem to have been dropped from the programme with no discussion. I was keen on it in principal but am completely disillusioned with the process. No one has made any effort to help me use the system, I appreciate I do not have a high level of IT skills but this should be taken into consideration otherwise .. will be skewed towards a subgroup of clinicians. **[No delays- free text qualitative data]**
You need to… they say to us, you need to give us the tools to be able to do this. So how do we get better internet connections? How can we have smartphones? Do you want to give us all iPad's, for example? **[Baseline 2012 E-HIT, C3 Interview 12]**

Public and Consumer Readiness
**Digital Literacy and Access**
"…because quite a few of them had *no digital knowledge whatsoever*, they had no access, they had nobody, that was able to show people how to use digital stuff …"– **[Digital Champions Interviews, March 2015]**<br><br>"…they give us these, and they said "*here you are here these tablets*", now the first we had ever seen a tablet was you took it with water and you put it in your mouth………..". **["House of Memories" Focus Group, March 2015]**
"The 20 to 30 year old market are used to using Wikipedia and Facebook and other things but the over 50s were very cautious and actually we've now scoped that back….". **[Final eHIT C1 Interview 1]**
"Not suitable for all patient groups ie elderly, those with poor IT skills or no access to IT hardware." **[No delays –free text qualitative data]**
"…there are lots of technically or digitally disadvantaged people in the city and I think for them the idea of technology in the home is something very futuristic." **[e-HIT Midpoint C2 Interview 7]**
**Agency & Lifestyle**
"….People don't prioritise health, *so if you are economically deprived*, what you prioritise is feelings of physical safety and financial safety so you could be worried about paying your rent, keeping the debt collectors off the door, anti-social behaviour in your neighbourhood. If you're more economically active then other things are a priority, ah, holidays, kids, schooling, housing, or next house, mortgage. " **[C2 implementer interview, June 2015]**
"….no problem in finding any number of Carers who and not just carers, people who wouldn't identify themselves as carers, there is people who have been worried about their parents, [……] and…..actually getting parents to sign up for this is a different story altogether. Right so how having, having my Mum, so me being worried about my Mum and being quite prepared to you know get a Service and you know pay £20.00 a month or more for that Service is relatively easy so getting my Mum to accept this when she is actually still quite well *and doesn't want people, doesn't necessarily want her children spying on her is quite another…*" **[C4, implementer interview June 2015]**

"We're trying to get people into eRedbook when they're shattered…., sort of, getting used to being a parent. It would be really useful, probably, to go back a bit later when actually things...one thing that we've been thinking about a lot **lately is the timing because** we know that that is a massive... has a massive impact on whether people decide to choose to do it or not, and what... our thinking around it now is that it really needs to be set up at antenatal stage when people are nesting and they're... they've got time. Then it's activated by the midwife in the hospital when there's a live birth and an NHS number to actually attach to that record, which means then that there's an Identifier and there's no mistake that that record is for that child." **–** **[Focus Group, April 2015]**
".. The only people that I can - *hand on heart* - say I haven't offered it to since we started to do this have been a couple of my Polish clients that haven't spoken English, the vast majority of them speak enough and if they speak and if they can understand me in the booking I will say would you like to access this and quite often if it's spoken they can but those, I have had a couple that have come and there is just not a word of English and for that reason I have chosen not to go there. **[Community midwife, Focus Group, April 2015]**
**Security & Trust**
"….I think the perception of risk to patient data is a big challenge so people, people are uncertain about the implications of sharing their data with an online system and they don't really, well it's difficult to explain the subtleties of the distinction between personally held record which they own the data and some kind of based system where it's you know so getting that, the messaging is really, really important so while we understand the concept of anonymising data and who will be able to identify patients from the data and who owns consent and everything else those are quite complicated messages to pass to the general public." **[Industry representative, C4 Final e-HIT Interview 3]**
"It's one of the challenges to moving the initiative forward. There's issues we're working on within our programme in terms of data transfer from tele-health to tele-care records, then from tele-care records into the private domain. The incoming challenge is, particularly from health practitioners, around how secure is the information, especially if patients start to hold the information themselves." **[Baseline e-HIT C2 2012 Interview 3]** <br>"….what our experience brought us to realise is that people will only use a kind of personal health journal and plan around serious conditions or long term conditions if it's something they can engage with their clinicians on and that was kind of a back to front approach. You're not really going to persuade people to go out and buy it as a consumer product if it's not something that there clinicians will engage with them on and look at and share the information that they've been collecting. **–** **[C3, implementer interview, June 2015]**
